# Appraisal of Cardiovascular Risk Factors, Biomarkers, and Ocular Imaging in Cardiovascular Risk Prediction

**DOI:** 10.2174/1573403X19666230727101926

**Published:** 2023-10-02

**Authors:** Julie S. Moore, M. Andrew Nesbit, Tara Moore

**Affiliations:** 1 School of Biomedical Sciences, Ulster University, York St, Belfast BT15 1ED, United Kingdom;; 2 Integrated Diagnostics Laboratory, Ulster University, 3-5a Frederick St, Belfast, Northern Ireland, United Kingdom

**Keywords:** Cardiovascular disease, risk factors, eye, microcirculation, biomarkers, medical imaging

## Abstract

Cardiovascular disease remains a leading cause of death worldwide despite the use of available cardiovascular disease risk prediction tools. Identification of high-risk individuals *via* risk stratification and screening at sub-clinical stages, which may be offered by ocular screening, is important to prevent major adverse cardiac events. Retinal microvasculature has been widely researched for potential application in both diabetes and cardiovascular disease risk prediction. However, the conjunctival microvasculature as a tool for cardiovascular disease risk prediction remains largely unexplored. The purpose of this review is to evaluate the current cardiovascular risk assessment methods, identifying gaps in the literature that imaging of the ocular microcirculation may have the potential to fill. This review also explores the themes of machine learning, risk scores, biomarkers, medical imaging, and clinical risk factors. Cardiovascular risk classification varies based on the population assessed, the risk factors included, and the assessment methods. A more tailored, standardised and feasible approach to cardiovascular risk prediction that utilises technological and medical imaging advances, which may be offered by ocular imaging, is required to support cardiovascular disease prevention strategies and clinical guidelines.

## 1 INTRODUCTION

Currently, an estimated 23% of all deaths in Northern Ireland are attributed to cardiovascular disease [1]. The British Heart Foundation (BHF) estimates that in Northern Ireland alone, cardiovascular disease costs the National Health Service £412 million. The aging population means that the burden of cardiovascular disease will only be exacerbated [2]. Prevention is better than cure, and hence critical review of current cardiovascular risk assessments is required. Application or addition of non-invasive vessel measurements of the ocular microcirculation, as demonstrated in the study by Brennan *et al.* (2019), may aid the personalisation of a risk score, as well as provide real-time data on the microvasculature [3]. Additionally, such advancements in technology and medical imaging should strengthen efforts to reduce morbidity and mortality rates associated with cardiovascular disease.

Cardiovascular risk assessment should support sub-clinical identification, stratification and management of individuals at risk of cardiovascular disease. However, a recent study pinpoints weaknesses, such as racial and ethnic disparities within current risk profiling methods, despite advances in healthcare [4]. In addition, existing cardiovascular disease risk scores are heterogenous and deficient in the vasculature and haemodynamic investigation, with reports of wide variation of risk classification [5]. Inaccuracies of cardiovascular disease risk scores may lead to a further burden on an already over-stretched health service, either through over or under-treating patients based on their risk score. A critical review of current cardiovascular risk assessment is required to evaluate areas of improvement and development and ultimately, help to reduce the burden of cardiovascular disease. The purpose of this review is to appraise current cardiovascular risk prediction methods, including machine learning, risk scores, biomarkers, medical imaging, and clinical risk factors. Moreover, this review aims to highlight the potential of ocular microcirculatory measurements for application in cardiovascular risk stratification.

## 2 OCULAR MEASUREMENTS IN DISEASE

The eye and the heart share many pathological risk factors, such as hypertension, hyperlipidemia, diabetes, and aging. Early identification of both eyes and cardiovascular pathology is essential, as in the example of hypertensive retinopathy (often characterised by cotton wool spots), a progressive loss of vision may occur.

Additionally, age-related macular degeneration and diabetic neuropathy are associated with atherosclerosis of the carotid artery and hence cerebral ischemia.

The microcirculation of the conjunctiva has been largely unexplored in comparison to that of the retina. Table **1** compares and summarises the advantages and disadvantages of two methods of microvascular examination, retinal and conjunctival. The retinal examination allows for detailed examination of blood vessels and can detect diabetic retinopathy but requires pupil dilation and specialised training for imaging and analysis. In contrast, conjunctival examination allows for easy and non-invasive visualisation of the blood vessels at the front of the eye, which can be imaged with simple equipment and does not require the use of ionising radiation or eye drops to dilate the pupil but may not be able to visualise certain structures, such as the optic disc that can help with arteriole and venule classification. The development of an innovative conjunctival microcirculation imaging technique, as we have proposed, may also be a promising prognostic tool to sub-clinically assess cardiovascular risk [3, 6, 7].

## 3 MACHINE LEARNING

The advances and applications of technology, such as with electronic healthcare records, as well as approaches to big data, have led to the development of automated and machine-learning-based techniques. Machine learning techniques are now front-runners in the search for earlier and more accurate cardiovascular risk stratification tools. Suri *et al.* (2022) reported a reduced risk of bias with machine learning compared to non-machine learning techniques for the risk estimation of cardiovascular disease [8]. Machine learning methods, such as neural networks, may enhance and help to personalise current risk scores [9, 10]. The area under the curve (AUC) for the studies that evaluated the use of machine learning for cardiovascular risk classification in this review ranged from 0.55 [11] with a Cox regression model to 0.92 [12] with a support vector machine approach. Table S1 summarises the literature on the role of machine learning in cardiovascular disease risk prediction. Table S1 also identifies the cardiovascular risk factors assessed throughout the literature. Age appeared to be the most commonly assessed risk factor, followed by gender, smoking status, total cholesterol, high-density lipoprotein, diabetes status, and blood pressure. The studies mentioned in Table **S1** include prospective studies and case-control studies [13-19]. Machine learning approaches are typically unbiased and more reproducible in comparison to more manual and subjective assessments. The machine learning models evaluated, particularly the Autoprognosis [13], and deep learning models, such as Deepsurv [15], were able to augment risk prediction compared to the traditional risk factors and conventional models, such as the Framingham Risk Score.

A similar binary logistic regression approach has been implemented with an algorithm based on conjunctival haemodynamic parameters (blood velocity) to support coronary artery disease screening [20]. The study by Awuah *et al.* demonstrated how assessment of the conjunctival microcirculation may be easily integrated into machine learning. Currently, there is no widely used risk score that utilises ocular measurements, such as that of the conjunctival microcirculation.

## 4 CURRENT CARDIOVASCULAR RISK SCORES: STRENGTHS AND WEAKNESSES

Awuah *et al*. [20] assessed ocular parameters to include conjunctival vessel diameter, cross sectional velocity, axial velocity, blood flow rate and wall shear rate, alongside clinical/lifestyle characteristics and blood biomarkers to include following lipids and markers of inflammation and endothelial dysfunction: haemoglobin A1c, sodium, potassium, urea, creatinine, creatinine clearance, haemoglobin, haematocrit, white cell count, platelet count, mean corpuscular volume, C-reactive protein, N-terminal pro-brain natriuretic peptide, total cholesterol, triglyceride, high density lipoprotein (HDL), low density lipoprotein, non-HDL, cholesterol HDL ratio, prothrombin time, activated partial thromboplastin clotting time, fibrinogen, urate, apolipoprotein A, apolipoprotein B, methylenetetrahydrofolate reductase, folic acid, homocysteine, vitamin B12, adiponectin, heart-type fatty acid binding protein, HDL3, interleukin (IL) -α, IL-β, IL-2, IL-4, IL-6, IL-8, IL-10, monocyte chemoattractant protein-1, tumor necrosis factor-α, vascular endothelial growth factor, interferon-γ, epidermal growth factor, asymmetric dimethylarginine, and leucine-rich α-2-glycoprotein-1. Consequently, the best classification variables identified through binary logistic regression analysis for high-risk cardiovascular disease patients versus controls were conjunctival cross-sectional velocity, N-terminal pro-brain natriuretic peptide, and adiponectin. The variables were employed within the algorithm-derived patient score and performed better than the QRISK3 score for the classification of high-risk cardiovascular disease patients compared to controls (Wilcoxon test p<2.2e^-16^ for the algorithm-derived patient score versus p=0.00031 for QRISK3). The application or addition of ocular measurements, such as conjunctival blood velocity to existing cardiovascular risk scores, may positively impact cardiovascular risk assessment.

Guidelines, such as those proposed by the National Institute of Clinical Excellence (NICE) in 2016 [21] or the European Society of Cardiology (ESC) [22], largely advocate the application of risk scores, such as QRISK3, for patient management. However, risk scores must evolve over time in response to trends in populations and various risk factors. The long-term Framingham risk study demonstrated this concept as it comprised three generations of participants with the Framingham off-spring study [23]. Although the Framingham risk study has been widely published and evaluated, it focused on a specific population from the United States. It may not comprehensively account for factors, such as ethnicity or socioeconomic status.

Ko *et al.* (2020) studied a cohort of 84,617 multi-ethnic Canadian participants with a maximum follow-up period of 5 years [24]. The predicted event rate was overestimated by 101% with the Framingham risk score. Similarly, Rospleszcz *et al.* (2019) also found the Framingham risk score overestimated cardiovascular disease risk [25]. However, van Kempen *et al.* (2014) found that this risk score performed well in the low-intermediate risk groups but poorly in the high-risk group [26]. Using data from the Framingham heart study, Zhang and Pincus (2015) found a 10% variation in the future lifespan of 28-38-year old’s, with blood pressure, blood glucose, weight and body mass index (BMI) being the most relevant factors in determining lifespan [27]. Various risk factors have been discussed in subsequent studies, such as in the example of the study by Esteghamati *et al.* (2013), which suggested that the waist-to-hip ratio is a superior predictor variable compared to BMI [28].

The prospective study by Maas *et al.* (2017) investigated plasma nitrate levels within the Framingham off-spring study [29]. The study found that while elevated plasma nitrate concentrations were associated with all-cause mortality, they were not associated with incident cardiovascular disease. Puurunen *et al.* (2018) also assessed the Framingham Heart Study participants and found that the blood biomarker of platelet function increased the risk of thrombosis associated with hyperplatelet aggregability to adenosine diphosphate (ADP) [30]. Lastly, two further biomarkers were assessed in the study by Lyngbæk *et al.* (2013), soluble urokinase plasminogen activator receptor (suPAR) and C-reactive protein (CRP) [31]. Lyngbæk *et al.* found that suPAR and CRP were significantly increased in participants who experienced a cardiovascular disease event compared to those who experienced no event (suPAR; 3.93 ng/mL (95% confidence intervals= 2.61-6.48) *vs.* 4.53 ng/mL (2.86-7.86), CRP; 1.61 mg/L (0.31-11.1) *vs.* 2.63 mg/L (0.40-13.8), respectively, p<0.0001).

The ESC introduced the SCORE cardiovascular risk assessment. Graversen *et al.* (2016) found that the SCORE algorithm predicted the risk of cardiovascular disease with an area under the curve (AUC) of 0.837, whereas in the original SCORE population, the AUC was 0.81 for the high-risk population and 0.74 for the low-risk population [32]. Improvements in SCORE have been suggested, such as by adding education level [33]. The study by Woźnicka-Leśkiewicz *et al.* (2015) concluded that the ankle-brachial index (ABI) and pulse wave velocity (PWV) in predicting cardiovascular disease risk according to the SCORE scale were more precise than that of the Framingham [34]. Contrastingly to the aforementioned studies, Woźnicka-Leśkiewicz *et al.* suggested that the Framingham scale underestimated those at high risk of cardiovascular disease.

The European Heart Journal (2021) introduced the SCORE2 and SCORE2-OP algorithms [35]. Compared to the SCORE algorithm, SCORE2 significantly improved the overall risk discrimination (p<0.001). Following the development of the SCORE2 algorithm, the SCORE2-OP model was established. The Harrell’s C-statistic (similar to AUC) performed better for SCORE-OP when compared to the atherosclerotic cardiovascular disease (ASCVD) score for all cohorts to Atherosclerosis Risk in Communities (ARIC) (0.644 *vs.* 0.668), MESA (0.645 *vs.* 0.654) and pooled trial populations (0.612 *vs.* 0.632), except for the CPRD cohort (0.663 *vs.* 0.657).

The concept of modifying and updating risk scores may enable a more accurate, as well as a more tailored risk score to be developed. This concept was demonstrated in a study by Argyridou *et al.* (2020) that modified SCORE to include walking pace, and the results showed improved performance metrics [36]. Correspondingly, the exercise capacity assessed *via* metabolic equivalents (METS) of a task was reported to be a valuable cardiovascular disease risk factor in the study by Salokari *et al.* (2019) [37], and as the walking pace increases, it would also be expected that METS would also increase [38].

Hippisley-Cox *et al.* (2017) first proposed QRISK3, the succeeding algorithm to the QRISK2 calculator [39]. This QRISK3 model considered the additional factors of chronic kidney disease (CKD), systolic blood pressure variability, migraine, corticosteroids, systemic lupus erythematosus (SLE), atypical antipsychotics, severe mental illness, and erectile dysfunction. The QRISK3 model with systolic blood pressure variability (R^2^ (%) = 59.6 (59.3 to 60.0) for women and 55.0 (54.6 to 55.3) for men) performed better than without the systolic blood pressure variability (59.5 (59.2 to 59.9) for women *vs.* 54.8 (54.5 to 55.2) for men), as well as performing better than the QRISK2 model (59.6 (59.2 to 60.0) for women *vs.* 54.8 (54.4 to 55.1) for men). However, in a more recent population cohort study by Livingstone *et al.* (2022), both CRISK and CRISK-CCI outperformed QRISK3 [40].

QRISK3 provides a 10-year risk score, yet the study by Wickramasinghe *et al.* (2014) indicated that the 10-year risk assessments might not comprehensively assess the cardiovascular disease burden of younger individuals and instead considered a long-term risk prediction tool of 30 years [41]. The study conducted by Chiuve *et al.* (2014) focused on minimising long-term cardiovascular disease risk through a Bayes information criterion- derived ‘Healthy Heart Risk Score’, proposed for use as a public health tool [42]. This risk score had a Harrell’s C-index of 0.72, demonstrating good discrimination. The risk score incorporated the familiar factors of age, smoking status, body mass index, physical activity levels, alcohol intake as well as diet through a composite diet score. Baik *et al.* (2012) and Georgousopoulou *et al.* (2020) suggested a similar incorporation of dietary evaluation for cardiovascular disease risk prediction [43, 44]. A summary of the commonly used risk scores described above, along with the risk factors they assess, is presented in Table **2**.

The above risk scores do not fully consider measurements of the vasculature. A major advantage of the conjunctival vessel measurements is the ability to objectively assess the conjunctival vessels and haemodynamics non-invasively, serially and without ionising radiation or the need for expensive equipment. Large longitudinal studies on the population would be required, particularly to assess the ability of these measurements, alongside other risk factors, to indicate long-term risk.

## 5 BIOMARKERS

Corbacho-Alonso *et al.* (2020), Racis *et al.* (2020), and Xuan *et al.* (2018) suggested blood or urine markers of oxidative stress as an addition to cardiovascular risk prediction models [45-47]. Other studies suggested blood biomarkers of inflammation [48, 49] or subclinical myocardial injury [50]. Repeat measurements of some biomarkers, such as high-sensitivity cardiac troponins, have been independently associated with cardiovascular events [51], while Ohm *et al.* [52] reported that recurrent cardiovascular events, including myocardial infarction or stroke, cannot be predicted by blood lipid levels. Despite the findings presented by Ohm *et al*., Willeit *et al.* [53] suggested that lipoprotein(a) may be a valuable addition to cardiovascular risk scores, such as the Framingham or Reynolds Risk Score. Conversely, Kouvari *et al.* (2019) implied that lipoprotein(a) testing may have less utility for women compared to men [54]. The literature largely agrees on the addition of such biomarker testing to cardiovascular risk prediction. Often the feasibility of adding clinical or laboratory-based measures may inhibit such cardiovascular risk assessments in individual or resource-limited settings within areas of deprivation, where often cardiovascular disease is most prevalent [55, 56]. Contrastingly, examination of the ocular microcirculation, as proposed within this review, would be inexpensive and require minimal training.

The conjunctival imaging system utilised by Brennan *et al.* [3] and the potential clinical applications of the imaging system are shown in Fig. (**1**). Brennan *et al*. described how ocular imaging could be performed with a slit lamp and a smartphone to record videos of conjunctival haemodynamics. The videos are then processed using a bespoke application, and ocular biomarkers measured include vessel diameter, cross-sectional velocity, axial velocity, blood flow rate, and wall shear rate. Brennan *et al.* later assessed these values for different patient cohorts, including myocardial infarction [6] and cyanotic congenital heart disease patients [7], reporting reductions in conjunctival vessel parameters, including axial velocity and wall shear rate, compared to control subjects.

Further alternatives to the traditional and clinical risk scores described in this review have been suggested through applications of multi-omics. Proteomics was shown to enrich cardiovascular disease risk stratification in multiple studies [45, 57, 58]. The study by Würtz *et al.* (2015) promoted the application of metabolomics through metabolic profiling for cardiovascular disease risk prevention [59]. Assessment of risk scores depends on the population studied, and hence, methods to advance the detection of individuals at high risk of cardiovascular disease may include epigenomics [60]. A genetic risk score based on 3 single-nucleotide polymorphisms was introduced in a study by Verbeek *et al.* (2019) and showed a positive association with plasma triglyceride levels, as well as an increased risk in cardiovascular disease [61]. Fig. (**2**) illustrates a selection of biomarkers associated with cardiovascular disease.

## 6 MEDICAL IMAGING AND CLINICAL RISK FACTORS

For participants with an ASCVD risk ≥10% in the prospective analysis conducted by Niu *et al.* (2020), the addition of electrocardiography (ECG) screening improved reclassification for those who did not experience events [62]. For low ASCVD-risk individuals, there was no significant association or improvement with the addition of ECG screening. Juxtaposing these findings, the earlier study by Goldman *et al.* (2019) suggested that ECG screening in low-risk individuals may improve cardiovascular disease risk stratification [63]. Badheka *et al.* (2013) suggested that the addition of ECG abnormalities to the Framingham Risk Score improves model discrimination and calibration [64].

Modification of blood pressure cut-offs failed to improve the discrimination of 10-year cardiovascular disease mortality in the study by Peng and Wang (2020) [65]. Likewise, blood pressure load was reported to not improve risk prediction based on 24-hour blood pressure levels [66]. However, the study also suggested that modification of blood pressure cut-offs may still be beneficial in the calibration and classification of high cardiovascular disease risk. Additionally, Bell *et al.* (2012) suggested that averaging 2 measurements of blood pressure and lipid biomarkers (total cholesterol and HDL) markedly improves overall cardiovascular risk prediction [67].

Systolic blood pressure variability was added to the QRISK3 score; however, Stevens *et al.* (2019) inferred that despite blood pressure variability being significant in a large dataset, it did not conclusively improve the performance of cardiovascular risk scores in the validation dataset [68]. Similarly, Ayala Solares *et al.* (2019) and Pool *et al.* (2018) found that using multiple blood pressure recordings from patients’ electronic healthcare records had negligible effects on models used to predict cardiovascular disease; also, using multiple blood pressure recordings resulted in a stronger association with incident cardiovascular disease than single measurements [69, 70]. Interestingly, findings of the SEPHARII study by Darabont *et al.* (2013) found that visit-to-visit systolic blood pressure variability strongly correlated with arterial stiffness, and this combination may strengthen cardiovascular risk prediction [71]. Said *et al.* (2018) also suggested arterial index as a predictor variable alongside pulse pressure to improve cardiovascular disease risk prediction [72]. Inflammation and hemostasis blood biomarkers alongside arterial stiffness were again found to support cardiovascular disease risk stratification in another study by Arnold *et al.* [73].

The ankle-brachial index (ABI) has also been proposed by previous studies to be a potential addition to cardiovascular disease risk prediction tools. A study by Fores *et al.* (2018) is one such study that supports ABI as a tool for cardiovascular disease risk stratification, with results of the ARTPER cohort suggesting that an ABI <0.9 is associated with an increased cardiovascular disease risk [74]. The study demonstrated improvements in the REGICOR scale and the Framingham Risk Score following the addition of ABI.

Flow-mediated dilation (FMD) is a measurement also considered in relation to cardiovascular disease risk. Irace *et al.* (2014) investigated FMD and found delayed vasodilation to be associated with increased cardiovascular disease risk [75]. Similarly, vascular functional robustness was propositioned by Kraushaar *et al.* (2018) as a potential biomarker for cardiovascular disease risk prediction [76].

Pulse wave analysis is a method suggested to assess potential biomarkers of cardiovascular disease risk. Results of the investigations by Cheng *et al.* (2016) found the systolic and diastolic rate constant for the central arterial pressure waveforms from pulse wave analysis to be valuable parameters for cardiovascular risk stratification [77]. A multi-marker approach was suggested by Greve *et al.* (2015) that utilises the presence of atherosclerotic plaque alongside albuminuria [78]. In this study, carotid-femoral pulse wave velocity was not found to support cardiovascular risk stratification. Berard *et al.* (2020) also reported classification improvements by including the number of carotid or femoral atherosclerotic plaques within cardiovascular disease risk prediction [79].

Van der Aalst *et al*. (2020) showed, through the ROBINSCA trial, that when compared with SCORE, the coronary artery calcium (CAC) scoring classed significantly fewer subjects as high risk [80]. The study highlights weaknesses of SCORE (such as lack of adaption to different ethnic or age groups) and suggests that CAC may be better for identifying patients who would benefit most from preventative treatment. A similar conclusion was reached in the study by Rana *et al.* (2012) that reported improvements in cardiovascular disease risk classification with CAC alone [81]. Craiem *et al.* (2020) also found that an increased CAC was associated with a higher cardiovascular disease risk, and that further details, such as calcification size and density, also improved the risk classification [82]. Yano *et al.* (2017) further conveyed that CAC may be an alternative to age in cardiovascular disease risk prediction [83]. However, Stigall-Weikle *et al.* (2022) argued that the use of ionising radiation might not be justified for risk prediction, particularly for individuals who are asymptomatic, and alternatively recommend non-invasive methods [84]. Like the proposed ocular vessel imaging, it could be argued that there may be more inexpensive and easily administered methods of assessing vasculature in cardiovascular disease risk prediction. The novel medical imaging methods of visualising the ocular microcirculation, as previously discussed in the study by Brennan *et al*. [3], were updated to permit automated 4K resolution video processing in the study by Jing *et al.* [85]. Future research should encompass such techniques for application in telemedicine.

A diagram summarising a selection of medical imaging techniques discussed in this review is shown in Fig. (**3**).

Standardised yet individualised risk assessments may support screening and augment patient care pathways. The addition of easily measured and interpreted vascular measurements to cardiovascular disease risk assessments, such as those proposed with the screening of conjunctival vasculature, may be beneficial. Refinement of practical multi-marker algorithms or machine learning approaches incorporating lifestyle, family history, clinical history, biomarkers and medical imaging results may be beneficial to support clinical decision-making. Future risk prediction scores should also be more accessible and encourage patients to actively make positive lifestyle changes where possible.

## Figures and Tables

**Fig. (1):**
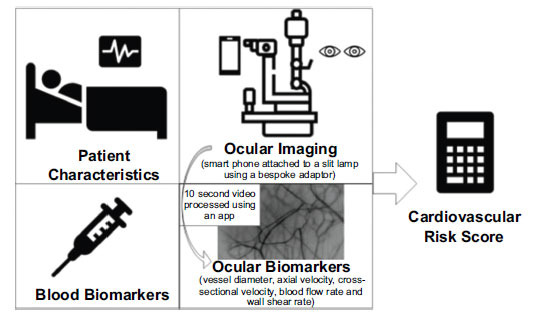
The conjunctival imaging system and its potential clinical applications.

**Fig. (2):**
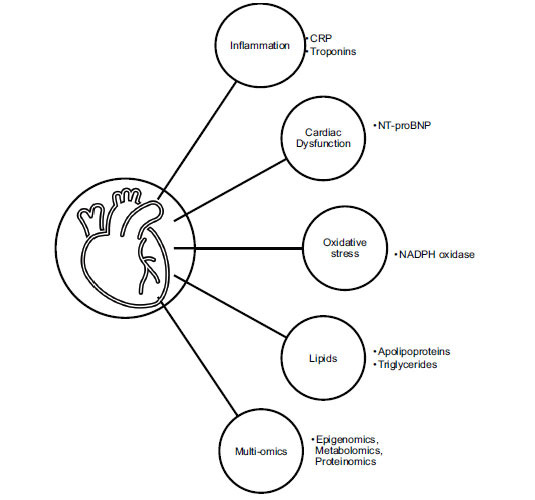
Biomarkers associated with cardiovascular disease. **Abbreviations:** CRP= C-Reactive Protein; NT-proBNP= N-Terminal pro B-type Natriuretic Peptide; NADPH= Nicotinamide Adenine Dinucleotide Phosphate.

**Fig. (3):**
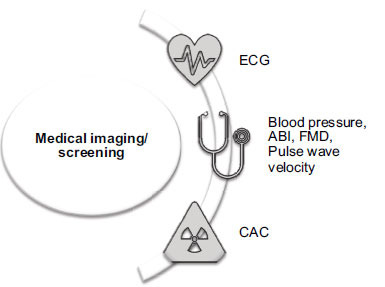
Medical imaging and clinical risk factors. ECG= Electrocardiogram, ABI= Ankle Brachial Index, FMD= Flow Mediated Dilation and CAC= Coronary Artery Calcium.

**Table 1:** Advantages and disadvantages of retinal versus microvascular examination in cardiovascular disease risk assessment.

	**Advantages**	**Disadvantages**
Retinal microvascular examination	Blood vessels examined for diabetic retinopathy Age-related macular degeneration may be assessed	Requires dilation of the pupil Specialised training is often required for imaging and analysis (*e.g*., optical coherence tomography training)
Conjunctival microvascular examination	Blood vessels can be easily and non-invasively seen at the front of the eye in contrast with the white background of the sclera Can be imaged inexpensively with minimal training using simple equipment	Inability to visualise certain structures within the eye, such as the optic disc that can help arteriole and venule classification

**Table 2:** Risk scores.

**Risk Score**	**Summary**
Framingham	Long-term multigenerational study on over 15,000 individuals spanning over 70 years. Risk factors include: • Blood pressure • Blood lipid levels • Age • Gender • Anthropometrics • Psychosocial factors • Smoking status
SCORE	ESC recommended risk score. Risk factors include: • Gender • Age • Cholesterol • Smoking status • Blood pressure
QRISK3	NICE recommended risk score. Risk factors include: • Age Gender • Ethnicity • Postcode • Family history of cardiovascular disease • CKD, atrial fibrillation, migraine, rheumatoid arthritis, SLE, severe mental illness • Medications Cholesterol/High-Density Lipoprotein (HDL) ratio • Blood pressure and systolic blood pressure variability • BMI

## LIST OF ABBREVIATIONS

ACC/AHA: American College of Cardiology/ American Heart Association
ANN: Artificial Neural Network
AUC-ROC: Area Under the Curve- Receiver Operating Characteristic curve
CI: Confidence Intervals
CVD: Cardiovascular Disease
FEq: Framingham Equation
K-NN: K-Nearest Neighbour algorithm
LRA: Logistic Regression Analysis
MAE: Major Adverse Event
ML: Machine Leaning
NPV: Negative Predictive Value
NRI: Neural Relational Inference
PH: Proportional Hazards
PPV: Positive Predictive Value
SVM: Support Vector Machines
TN: True Negative
TP: True Positive
