# Supplementary material for: Appraisal of Cardiovascular Risk Factors, Biomarkers, and Ocular Imaging in Cardiovascular Risk Prediction
Source: Curr Cardiol Rev. 2023 Oct 2;19(6):e270723219181. doi: 10.2174/1573403X19666230727101926 (PMC10636798; doi:10.2174/1573403X19666230727101926)
Supplement: Supplementary file 1 — Supplementary material is available on the publisher’s website along with the published article. [file CCR-19-E270723219181_SD1.pdf]

Supplementary Material

Appraisal of Cardiovascular Risk Factors, Biomarkers, and Ocular Imaging in Cardiovascular Risk Prediction

Julie S. Moore[1,2,*], M. Andrew Nesbit[1,2] and Tara Moore[1,2]

[1]*School of Biomedical Sciences, Ulster University, UK;* [2]*Integrated Diagnostics Laboratory, Ulster University, 3-5a Frederick St, Belfast, Northern Ireland, United Kingdom*

Table S1. Machine learning methods.

Study	Design	Aims	Risk Factors Assessed	Key Findings
Alaa *et al.* 2019 [13]	A prospective study on 423,604 UK Biobank participants.	The study aims to assess the potential value of using ML approaches to derive risk prediction models for CVD.	473 variables were assessed. Physical activity (usual walking pace) and information on blood measurements were the top predictor variables.	AutoPrognosis model improved risk prediction (AUC-ROC: 0.774, 95% CI: 0.768-0.780) compared to Framingham score (AUC-ROC: 0.724, 95% CI: 0.720-0.728, p < 0.001), Cox PH model with conventional risk factors (AUC-ROC: 0.734, 95% CI: 0.729-0.739, p < 0.001), and Cox PH model with all UK Biobank variables (AUC-ROC: 0.758, 95% CI: 0.753-0.763, p < 0.001). Out of 4,801 CVD cases recorded within 5 years of baseline, AutoPrognosis was able to correctly predict 368 more cases compared to the Framingham score.
Dimopoulos *et al.* 2018 [14]	10-year ATTICA prospective study (n=2020 adults)	To explore the potential of using machine learning methodologies on cardiovascular disease prediction, especially compared to an established risk tool, the HellenicSCORE.	A total of 16 variables were assessed, including age, sex, smoking status, systolic blood pressure, and total cholesterol levels.	HellenicSCORE showed an accuracy of 85%, specificity of 20%, sensitivity of 97%, PPV of 87%, and NPV value of 58%, whereas for the ML methodologies, accuracy ranged from 65 to 84%, specificity from 46 to 56%, sensitivity from 67 to 89%, PPV from 89 to 91%, and NPV from 24 to 45%; random forest gave the best results, while the k-NN gave the poorest results.
Hathaway *et al.* 2021 [15]	6814 participants from the Multi-Ethnic Study of Atherosclerosis (MESA) were followed over 16 years	To investigate whether novel deep learning survival models can augment atherosclerotic cardiovas-	A total of 33 variables were assessed, and age and coronary artery calcium score were	Compared to the COXPH model, DeepSurv significantly improved ASCVD risk prediction for MAE

		cular disease (ASCVD) risk prediction over existing statistical and machine learning approaches.	found to be the top predictor variables.	(AUC: 0.82 vs. 0.80, P ≤ 0.001) and mortality (AUC: 0.87 vs. 0.84, P ≤ 0.001) with traditional risk factors alone. Implementing non-categorical NRI, resulted in a >40% increase in correct reclassification compared to the COXPH model for both MAE and mortality (P ≤ 0.05). Assessing the relative risk of participants, DeepSurv was the only learning algorithm to develop significantly improved risk score criteria, which outcompeted COXPH for both MAE (4.22 vs. 3.61, P = 0.043) and mortality (6.81 vs. 5.52, P = 0.044). The addition of inflammatory or imaging biomarkers to traditional risk factors showed minimal/no significant improvement in model prediction.
Kakadiaris *et al.* 2018 [12]	Machine learning risk calculator was developed based on SVMs using a 13-year follow-up data set from MESA (the Multi-Ethnic Study of Atherosclerosis) of 6459 participants who were atherosclerotic CVD-free at baseline.	Using machine learning and the same risk factors used by ACC/AHA Risk Calculator, the study aimed to improve CVD risk stratification. The approach was tested in MESA (the Multi-Ethnic Study of Atherosclerosis) and also used FLEMENGHO (the Flemish Study on Environment, Genes and Health Outcomes) for external validation.	Age, gender, ethnicity, total cholesterol, high-density lipoprotein, systolic blood pressure, history of hypertension, history of diabetes and smoking history.	Sensitivity= 0.86, specificity= 0.95, and AUC= 0.92.
Mannan *et al.* 2013 [16]	The Framingham Heart Study Dataset was used.	The study aims at illustrating SAS computer programs for estimating over-optimism in measures of discrimination using two bootstrap validation algorithms through a concrete example from an active research field of chronic disease risk prediction–validation of a CVD risk prediction model.	Systolic and diastolic blood pressure, total and high-density lipoprotein cholesterol, smoking status, age, sex, diabetes status, triglycerides and body mass index.	The degree of over-optimism in both Harrell's C and Somers' D statistics was low. Both these statistics were corrected for over-optimism by subtracting over-optimism from their observed values. Between the two bootstrap validation algorithms, the degree of over-optimism was estimated to be higher for stepwise bootstrap validation.
Sajid *et al.* 2021 [17]	A case-control study	A total sample of 460, 230 cases and 230 matched controls were selected	Age groups, hypertension, low fruit consumption, smoking	The first finalised model (ANN with 1 hidden layer) provided a risk

		from September, 2018 to February, 2019.	history, low vegetable consumption, physical inactivity, red meat/poultry consumption, diabetes mellitus, consumption of high salty foods, abdominal obesity, high fried foods/ trans fats, parental history of cardiovascular disease and self-reported general stress.	prediction model with 81.09% accuracy and 0.871 AUC. The sensitivity (0.780) and specificity (0.848) values of the ANN-based model showed consistency in predicting the TP and TN values of the dataset. The linear SVM reported the best hyperplanes with an accuracy of the model of 80.86%.
Unnikrishnan *et al.* 2016 [18]	3654 participants over 15 years (5-year follow-up intervals) via the Blue Mountain Eye Study database	To determine if the parameters used by the Framingham model are relevant to a different database, this study measured the sensitivity and specificity obtained using SVM.	Age, body mass index, smoking status, gender, total cholesterol, systolic blood pressure, high-density lipoprotein cholesterol, diabetes status, hypertension medications, retinopathy and diastolic blood pressure.	The sensitivity obtained from the FEq was 0.52 (95% CI: 0.4096 to 0.6275), from the LRA, was 0.48 (95% CI: 0.3817 to 0.5809), and from the SVM was 0.682 (95% CI: 0.589 to 0.764). This shows that the sensitivity of the FEq and logistic analysis is comparable, while that of SVM is better and thus provides a better risk assessment. The AUC test shows that the SVM results were greatly improved (0.71) compared to Framingham (0.57) or LRA (0.63).
Unterhuber *et al.* 2021 [11]	Using the OLINK-Cardiovascular-II panel, 92 proteins were measured in a cohort of 1,998 individuals from the LIFE-Heart Study (derivation) and 772 subjects from the PLIC (Progressione della Lesione Intimale Carotidea) cohort (external validation).	This study compared proteomics-enabled ML algorithms with classical and clinical risk prediction methods for all-cause mortality in cohorts of patients with cardiovascular risk factors in the LIFE-Heart Study, followed by validation in the PLIC study.	Age, sex, body mass index, smoking status, diabetes, use of antihypertensive medication, high-density lipoprotein cholesterol, total cholesterol, and triglyceride levels and influencing proteins, including brain natriuretic peptide, tumour necrosis factor-related apoptosis-inducing ligand receptor 2 and chymotrypsin.	On internal and external validation, the Framingham Risk Score achieved AUCs of 0.64 (95% CI: 0.59-0.68) and 0.65 (95% CI: 0.58-0.74), logistic regression AUCs of 0.65 (95% CI: 0.57-0.73) and 0.67 (95% CI: 0.59-0.74), Cox regression AUCs of 0.55 (95% CI: 0.51–0.59) and 0.65 (95% CI: 0.57-0.73), the XGBoost classifier AUCs of 0.83 (95% CI: 0.79-0.87) and 0.91 (95% CI: 0.86-0.95), the XGBoost survival estimator AUCs of 0.83 (95% CI: 0.79-0.87) and 0.93 (95% CI: 0.88-0.97), and the neural network AUCs of 0.87 (95% CI: 0.83-0.91) and 0.94 (95% CI: 0.90-0.98), respectively (modern vs classical ML: $P < 0.001$).
Weng *et al.* 2017 [19]	Prospective cohort study (10 years) using routine clinical data of 378,256 patients from UK family practices, free from cardiovascular disease at the outset.	The aim of this study was to evaluate whether ML can improve the accuracy of cardiovascular risk prediction within a large	Gender, age, smoking status, systolic blood pressure, blood pressure treatment, total cholesterol, high-	Compared to the established risk prediction algorithm (AUC 0.728, 95% CI 0.723–0.735), ML algorithms improved

		general primary care population. The study also sought to determine which class of ML algorithm has the highest predictive accuracy.	density lipoprotein cholesterol and diabetes.	prediction: random forest +1.7% (AUC 0.745, 95% CI 0.739–0.750), logistic regression +3.2% (AUC 0.760, 95% CI 0.755–0.766), gradient boosting +3.3% (AUC 0.761, 95% CI 0.755–0.766), neural networks +3.6% (AUC 0.764, 95% CI 0.759–0.769). The highest achieving (neural networks) algorithm predicted 4,998/7,404 cases (sensitivity 67.5%, PPV 18.4%) and 53,458/75,585 non-cases (specificity 70.7%, NPV 95.7%), correctly predicting 355 (+7.6%) more patients who developed CVD compared to the established algorithm.

Abbreviations: ACC/AHA= American College of Cardiology/American Heart Association, ANN= Artificial Neural Network, AUC-ROC= Area Under the Curve- Receiver Operating Characteristic curve, CI= Confidence Intervals, CVD= Cardiovascular Disease, FEq= Framingham Equation, K-NN= K-Nearest Neighbour algorithm, LRA= Logistic Regression Analysis, MAE= Major Adverse Event, ML= Machine Leaning, NPV= Negative Predictive Value, NRI= Neural Relational Inference, PH= Proportional Hazards, PPV= Positive Predictive Value, SVM= Support Vector Machines, TN= True Negative, TP= True Positive
